# POLR3A variants with striatal involvement and extrapyramidal movement disorder

**DOI:** 10.1007/s10048-019-00602-4

**Published:** 2020-01-15

**Authors:** Inga Harting, Murtadha Al-Saady, Ingeborg Krägeloh-Mann, Annette Bley, Maja Hempel, Tatjana Bierhals, Stephanie Karch, Ute Moog, Geneviève Bernard, Richard Huntsman, Rosalina M. L. van Spaendonk, Maaike Vreeburg, Agustí Rodríguez-Palmero, Aurora Pujol, Marjo S. van der Knaap, Petra J. W. Pouwels, Nicole I. Wolf

**Affiliations:** 1 Department of Neuroradiology, University Hospital Heidelberg, Im Neuenheimer Feld 400, 69120 Heidelberg, Germany; 2 Department of Child Neurology, Center for Childhood White Matter Diseases, Emma Children’s Hospital, Vrije Universiteit, and Amsterdam Neuroscience, Amsterdam University Medical Centers, Amsterdam, The Netherlands; 3 Department of Paediatric Neurology and Developmental Medicine, University Children’s Hospital Tübingen, Tübingen, Germany; 4 University Children’s Hospital, University Medical Center Hamburg Eppendorf, Hamburg, Germany; 5 Institute of Human Genetics, University Medical Center Hamburg-Eppendorf, 20246 Hamburg, Germany; 6 Division of Neuropaediatrics and Metabolic Medicine, Centre for Child and Adolescent Medicine, Clinic I, University Hospital Heidelberg, Im Neuenheimer Feld 430, 69120 Heidelberg, Germany; 7 Institute of Human Genetics, Heidelberg University, Im Neuenheimer Feld 366, 69120 Heidelberg, Germany; 8 Departments of Neurology and Neurosurgery, Paediatrics and Human Genetics, Department of Specialized Medicine, Division of Medical Genetics, McGill University Health Center, and Child Health and Human Development Program, Research Institute of the McGill University Health Centre, McGill University, McGill University, Montreal, Quebec Canada; 9 Division of Paediatric Neurology, University of Saskatchewan, Saskatoon, Saskatchewan Canada; 10 Department of Clinical Genetics, Vrije Universiteit, Amsterdam University Medical Centers, Amsterdam, The Netherlands; 11 Department of Clinical Genetics, Maastricht University Medical Center, Maastricht, The Netherlands; 12 Neurometabolic Diseases Laboratory, Bellvitge Biomedical Research Institute (IDIBELL), Hospitalet de Llobregat, Barcelona, Catalonia Spain; 13 Department of Pediatrics, Paediatric Neurology Unit, University Hospital Germans Trias i Pujol, Badalona, Barcelona, Catalonia Spain; 14 Centre for Biomedical Research on Rare Diseases (CIBERER), Institute Carlos III, Madrid, Spain; 15 Catalan Institution for Research and Advanced Studies (ICREA), Barcelona, Spain; 16 Department of Functional Genomics, Center for Neurogenomics and Cognitive Research, Vrije Universiteit Amsterdam, Amsterdam, The Netherlands; 17 Department of Radiology and Nuclear Medicine, Vrije Universiteit, Amsterdam University Medical Centers, Amsterdam, The Netherlands

**Keywords:** *POLR3A*, MRI, Basal ganglia, Striatum, Superior cerebellar peduncle, Inferior cerebellar peduncle, Brainstem, Hypomyelination

## Abstract

Biallelic variants in *POLR3A* cause 4H leukodystrophy, characterized by hypomyelination in combination with cerebellar and pyramidal signs and variable non-neurological manifestations. Basal ganglia are spared in 4H leukodystrophy, and dystonia is not prominent. Three patients with variants in *POLR3A,* an atypical presentation with dystonia, and MR involvement of putamen and caudate nucleus (striatum) and red nucleus have previously been reported. Genetic, clinical findings and 18 MRI scans from nine patients with homozygous or compound heterozygous *POLR3A* variants and predominant striatal changes were retrospectively reviewed in order to characterize the striatal variant of *POLR3A*-associated disease. Prominent extrapyramidal involvement was the predominant clinical sign in all patients. The three youngest children were severely affected with muscle hypotonia, impaired head control, and choreic movements. Presentation of the six older patients was milder. Two brothers diagnosed with juvenile parkinsonism were homozygous for the c.1771-6C > G variant in *POLR3A*; the other seven either carried c.1771-6C > G (*n* = 1) or c.1771-7C > G (*n* = 7) together with another variant (missense, synonymous, or intronic). Striatal T2-hyperintensity and atrophy together with involvement of the superior cerebellar peduncles were characteristic. Additional MRI findings were involvement of dentate nuclei, hila, or peridentate white matter (3, 6, and 4/9), inferior cerebellar peduncles (6/9), red nuclei (2/9), and abnormal myelination of pyramidal and visual tracts (6/9) but no frank hypomyelination. Clinical and MRI findings in patients with a striatal variant of *POLR3A*-related disease are distinct from 4H leukodystrophy and associated with one of two intronic variants, c.1771-6C > G or c.1771-7C > G, in combination with another *POLR3A* variant.

**Electronic supplementary material:**

The online version of this article (10.1007/s10048-019-00602-4) contains supplementary material, which is available to authorized users.

## Introduction

RNA polymerase III (POLR3) transcribes genes encoding small, non-coding RNAs including tRNAs, 5S RNA, 7SK RNA, and U6 small nuclear RNA, which are involved in the regulation of transcription, RNA processing, and translation [1].

Disease-causing variants in genes coding for POLR3 subunits were first discovered in patients with hypomyelinating leukodystrophy. They are located in *POLR3A* [2] and *POLR3B* [1, 3], which encode the largest and second largest subunits of POLR3 forming the catalytic centre of the enzyme, as well as in *POLR1C* [4], a gene encoding a shared POLR1 and POLR3 subunit. The resulting 4H leukodystrophy (hypomyelination, hypodontia, hypogonadotropic hypogonadism) is characterized by hypomyelination in combination with early cerebellar and subsequent pyramidal signs (usually mild) and variable non-neurological manifestations, namely dental and endocrine features as well as myopia [5]. Ataxia is the predominant clinical finding in 4H leukodystrophy. Dystonia is an additional, common, and initially under-recognized feature in 4H leukodystrophy [6], but not prominent at disease onset, and basal ganglia abnormalities as a potential correlate of dystonia have not been reported in 4H leukodystrophy. Clinical manifestations and hypomyelination in 4H leukodystrophy are more severe in patients with variants in *POLR3A* and *POLR1C* than in patients with variants in *POLR3B* [7, 8]; hypomyelination, however, is not obligatory, and manifestation without hypomyelination occurs in patients with variants in *POLR3A* or *POLR3B* [9].

During the last years, *POLR3A* variants without predominant ataxia have been reported: A striatal manifestation with predominant dystonia and MR involvement of putamen, caudate and red nucleus due to a homozygous founder variant in intron 13 was reported for three patients from two families with a Roma background [10]. In addition, biallelic *POLR3A* variants have been recognized as a cause of hereditary spastic ataxia [11, 12].

In order to characterize the striatal variant of *POLR3A*-related disease, we reviewed clinical, genetic, and MRI findings of nine patients with *POLR3A* variants and striatal changes.

## Patients and methods

We retrospectively identified nine patients from eight families with biallelic *POLR3A* variants and striatal changes on MRI through the patient database at the Center for Childhood White Matter Disorders Amsterdam. Patients were referred to the Center for Childhood White Matter Disorders Amsterdam after identification of *POLR3A* variants, but without typical presentation for 4H leukodystrophy, for diagnostic evaluation. In all patients, *POLR3A* variants were identified by diagnostic whole exome sequencing, performed at different centres. Segregation analysis established their biallelic occurrence in all patients except patient 6, of whom only one parent was available for testing and carried one of the patient’s two variants. No other variants were found explaining the movement disorder. NIW saw patients 1, 3, 7–9; IKM saw patient 5, AB patient 2, GB and RH patient 6, AR-P patient 4. Records were reviewed for clinical presentation and are summarized in Table 1; for case histories, see supplemental material.

**Table 1:** Main clinical characteristics. *BEAR* brainstem evoked acoustic responses, *F* female, *M* male, *mo* month, *n/a* not applicable, *yrs.* years

	Patient 1	Patient 2	Patient 3	Patient 4	Patient 5	Patient 6	Patient 7	Patient 8	Patient 9
Gender	F	F	F	M	F	M	M	M	F
Current age	2 yrs	2 yrs	21 mo	7 yrs	7 yrs	6 yrs	22 yrs	26 yrs	29 yrs
Affected siblings	No	No	No	No	No	No	Yes, patient 8	Yes, patient 7	No
Consanguinity	No	No	No	No	No	No	Yes	Yes	No
Age at onset	2 months	First days of life	First days of life	First year of life	Second year of life	13 months	4 years	Second year of life	14 months
Signs/symptoms at onset	No smiling, failure to thrive	No crying, absent visual contact	Abnormal movements, restlessness	Global developmental delay, poor facial expression	Mild motor delay	Abnormal gait	Psychomotor retardation	Mild delay in language acquisition	Abnormal gait, prone to falling
Age at last examination	17 mo	2 yrs.	16 mo	5 yrs	5 yrs	4 yrs.	21 yrs	26 yrs	29 yrs
Axial hypotonia	Yes	Yes, if relaxed. Severe opisthotonus in agitated episodes	Severe	No	No	Mild	No	No	No
Head balance	Suboptimal	Suboptimal at 2 mo, lost at 4 mo	Suboptimal	Suboptimal at three mo	Normal	Normal	Normal	Normal	Normal
Ataxia	No intentional movements	No intentional movements	No	Yes	Mild gait ataxia	Yes	No	No	Head titubation
Pyramidal signs	No	Yes	No	Yes	No	Yes	No	No	Mild pyramidal signs (legs)
Extrapyramidal signs	Yes, choreic movements and opisthotonus	Yes, choreic movements	Yes, choreic movements and opisthotonus	Yes, dystonia and bradykinesia	No	Yes, dystonia	Severe, compatible with Parkinsonism; no tremor	Severe, compatible with Parkinsonism; in addition (rubral) tremor (3/s), increasing with action	Yes, (rubral) tremor (3/s), increasing with action; mild posturing
Eye movements	Saccadic pursuit	No fixation	Short periods of fixation	Normal	Normal	Saccadic pursuits and hypometric saccades	Saccadic pursuit	Saccadic pursuit and hypometric saccades	Saccadic pursuit
Highest motor achievement	Some head balance	Some head balance	Some head balance, tries to reach for objects	Walks with posterior walker, manages to walk 20 steps without support	Walking without support	Walking without support	Walking without support	Walking without support	Walking without support (age 14 mo), wheelchair dependent from age 12 yrs
Swallowing problems	Mild	Severe	Severe (nasogastric tube)	Yes, especially with liquids (prone to aspiration)	No	No	No	Severe dysphagia	Yes
Speech and language	None	None	None	Delayed (uses about 20 words, difficult to understand)	Mild delay in language development, at age 4 yrs., stutter	Speech delay, dysarthria	Severe dysarthria	Language deterioration from age 5 yrs., now anarthric	Severe dysarthria
Cognition	Severe global delay	Severe global delay assumed	Severe global delay	Not formally tested but seems normal	Mild learning difficulties	Not formally tested but seems normal	Learning disability	Learning disability	Normal
Epilepsy	Yes, myoclonic jerks from age 15 mo	No	No	No	No	No	No	No	No
Dentition	Abnormal (lack of maxillary incisors)	Abnormal (delayed dentition, deciduous molars first teeth to erupt)	Abnormal (lack of maxillary incisors)	Abnormal (delayed eruption of maxillary incisors)	Normal	Normal	Normal	Abnormal (first teeth to erupt maxillary incisors, 2 persisting decidual teeth)	Abnormal (molars first to erupt, incisors erupted at 4y of age)
Puberty development	n/a	n/a	n/a	n/a	n/a	n/a	Normal	Normal	Normal
Growth	Failure to thrive	Failure to thrive	Failure to thrive	Failure to thrive	Normal	n/a	Very low weight due to inadequate intake	Very low weight due to inadequate intake	Normal
Head circumference	Normal	Normal	Normal	Normal	Normal	Normal	Normal	Normal	Normal
Myopia	Not tested	Not tested	Not tested	No	Mild myopia	No	No	No	Mild myopia
Hearing loss	Not tested	Not tested	Abnormal BEAR	BEAR normal	Not tested, clinically normal	Not tested, clinically normal	Not tested, clinically normal	Not tested, clinically normal	Not tested, clinically normal
Other	n/a	Laboured breathing	Prone to respiratory tract infections; bacterial meningitis	n/a	n/a	n/a	n/a	n/a	n/a

The patients’ 18 cranial MRI scans (age at examination 0.5–29 years, mean 9.1 years, median 4.8 years) were systematically reviewed in consensus by a pediatric neuroradiologist (IH) and pediatric neurologist (NIW). Axial T2-weighted (T2w) and T1-weighted (T1w) images were available for all MRI scans, sagittal T1w images for all but the follow-up MRI in patient 6 (sagittal 3D-T2w), diffusion-weighted imaging (DWI) with apparent diffusion coefficient (ADC) for at least one MRI in all patients (13/18 MRIs). MRI was assessed for presence and extent of T2w grey and white matter changes, in particular for involvement of deep grey matter and brainstem tracts, and for corresponding T1-signal changes. DWI and ADC-maps were inspected for restricted diffusion, namely hyperintensity on DWI and corresponding low signal on ADC (below 60 × 10-5 mm2/s), or increased diffusion with high signal on ADC (above 100–110 × 10-5 mm2/s, for basal ganglia and white matter, respectively [13–15]).

T2 gradient echo and susceptibility-weighted images, available for patient 9 and first MRIs of patients 1, 4, 5, and 6 (field strength 1.5 (2) or 3 Tesla(3)), were checked for hypointensities due to calcifications and/or blood degradation products; the cerebral CT scan available for patient 8 was checked for hyperdensities. Spinal MRIs were available for patients 1, 5, and 6.

For comparison of involvement of cerebellar peduncles and/or striatum in typical 4H leukodystrophy, we additionally reviewed 40 MRIs of 36 patients with 4H leukodystrophy and imaging between 2.8 and 40 years previously published [7].

## Results

### Patients

All patients except patient 5 had an extrapyramidal movement disorder. Onset of symptoms varied between neonatal period (patients 1–3), infancy (patients 4–6), and early childhood (patients 7–9). Initial symptoms in the patients with neonatal presentation comprised abnormal choreic movements, restlessness, poor visual contact, failure to thrive due to swallowing difficulties, and severe global developmental delay. In the patients with infantile presentation, there were developmental delay more of motor than of cognitive development and extrapyramidal signs with dystonic posturing and poor facial expression (excepting patient 5 who had only mild ataxia). In the patients with early childhood presentation, initial motor development was normal: All patients walked without support age 12–15 months, although at least in patient 9 there were always concerns of frequent falls. In these patients, both motor function and expressive speech deteriorated in childhood with resulting severe dysarthria/anarthria and dysphagia. For a detailed description, see supplemental case reports and Table 1.

Dentition was abnormal in six of nine patients. Of six patients tested, two had mild myopia (patients 5 and 9). Only three patients (7–9) were old enough to exclude delayed puberty due to hypogonadotropic hypogonadism. Clinical, genetic, and MRI findings are summarized in Tables 1, 2, and 3 with patients sorted for age at first MRI.

**Table 2:** Genetic findings. Genetic variants for all patients

Patient	Variant 1	Variant 2
1	c.1771-7C > G p.(Glu548_Tyr637del) / p.(Pro591Metfs*9)	c.1048 + 5G > T p.(Glu350Glufs*27)
2	c.1771-7C > G p.(Glu548_Tyr637del) / p.(Pro591Metfs*9)	c.4025-1G > A p.?
3	c.1771-7C > G p.(Glu548_Tyr637del) / p.(Pro591Metfs*9)	c.2713G > A p.(Asp905Asn)
4	c.1771-7C > G p.(Glu548_Tyr637del) / p.(Pro591Metfs*9)	c.3387C > A p.(Leu1129Leu)
5	c.1771-7C > G p.(Glu548_Tyr637del) / p.(Pro591Metfs*9)	c.2809G > A p.(Glu937Lys)
6	c.1771-7C > G p.(Glu548_Tyr637del) / p.(Pro591Metfs*9)	c.1771-6C > G p.(Pro591Metfs*9)
7 & 8	c.1771-6C > G p.(Pro591Metfs*9)	c.1771-6C > G p.(Pro591Metfs*9)
9	c.1771-6C > G p.(Pro591Metfs*9)	c.2045G > A p.(Arg682Gln)

**Table 3:** Overview of MRI changes (sorted by age at first MRI). *ALIC* anterior limb of internal capsule, *CC* corpus callosum, *cor.rad*. corona radiate, *c.sem.* centrum semiovale, *decuss. SCP* decussation of superior cerebellar peduncles, *dent. ncl.* dentate nucleus, *front*. frontal, *hilus* hilus of dentate ncl., *ICP inferior cerebellar peduncles, myel. delay* myelination delay, *pall.* pallidum, *perident. wm* peridentate white matter, *pyr.tr.* pyramidal tract, *resid.* residual, *SCP* superior cerebellar peduncles, *subcort*. subcortical, *supratent. wm* supratentorial white matter, *temp*. temporal, *vol.* volume, *yrs.* years, *↑T2/↓T1* hyperintensity on T2//hypointensity on T1w images, *~* normal signal compared to controls

Patient	Age at MRI [yrs]	Basal ganglia		Brainstem/cerebellum							Supratent. wm			
		Striatum	Pall.	red ncl.	decuss. SCP	SCP	dent. ncl.	hilus	perident. wm	ICP	Abnormal	pyr. tract	Optic radiation	Other regions
1	0.5	~	~	~	↑T2, ↓T1	~	~	~	~	~	yes	myel. delay: subcort., c.sem., cor.rad.	myel. delay	~
	1.5	↑T2, ↓vol.	~	~	↑T2, ↓T1	↑T2, ↓T1	~	↑T2, ↓T1	↑T2, ↓T1	↑T2, ↓T1	yes	↑T2: subcort., c.sem., cor.rad.	new ↑T2	↑T2 c.sem., cor.rad. beyond pyr.tr., subcort. wm
2	0.9	↑T2, ↓vol.	~	↑T2	↑T2, ↓T1	↑T2, ↓T1	↑T2, ↓T1	↑T2, ↓T1	~	~	yes	mild ↑T2: subcort., c.sem., cor.rad.	mild ↑T2	↑T2 c.sem., cor. rad. beyond pyr. tr.
3	1.0	↑T2, ↓vol.	↓T2	~	↑T2, ↓T1	↑T2, ↓T1	↑T2, ↓T1	↑T2, ↓T1	↑T2, ↓T1	↑T2, ↓T1	yes	mild ↑T2: subcort., c.sem., cor.rad.	mild ↑T2	↑T2 c.sem., cor. rad. beyond pyr. tr.
4	1.7	↓vol.	~	~	↑T2, ↓T1	↑T2, ↓T1	↑T2, ↓T1	↑T2, ↓T1	↑T2, ↓T1	↑T2, ↓T1	yes	mild ↑T2: subcort., c.sem., cor.rad.	mild ↑T2	~
	2.9	↑T2, ↓vol.	↓T2	~	↑T2, ↓T1	↑T2, ↓T1	↑T2, ↓T1	↑T2, ↓T1	↑T2, ↓T1	↑T2, ↓T1, increasing	yes	mild ↑T2: subcort., c.sem., cor.rad.	incr. ↑T2	new ↑T2 ILF
5	2.0	~	↓T2	↑T2 (right)	↑T2, ↓T1	↑T2, ↓T1	~	↑T2, ↓T1	↑T2, ↓T1	↑T2, ↓T1	yes	~	mild ↑T2	non-specific subcort. ↑T2, front. & temp. wm
	4.8	↑T2, ↓vol.	↓T2	~	~	residual ↑T2	~	~	~	residual ↑T2	yes	~	mild ↑T2	non-specific subcort. ↑T2, front. & temp. wm
6	4.0	↓vol., mild↑T2	↓T2	~	~	↑T2, ↓T1	~	~	~	↑T2, ↓T1	no	~	~	~
	4.9	↓vol., mild↑T2	↓T2	~	~	↑T2, ↓T1	~	~	~	↑T2, ↓T1	no	~	~	~
7	9.9	↑T2, ↓vol.	~	~	~	↑T2	~	~	~	~	no	~	~	~
	13.6	↑T2, ↓vol.	~	~	↑T2	↑T2	~	~	~	~	no	~	~	~
	18.5	↑T2, ↓vol.	~	~	~	~	~	~	~	~	no	~	~	~
8	12.1	↑T2, ↓vol.	~	~	~	↑T2	~	↑T2, ↓T1	~	~	no	~	~	~
	14.9	↑T2, ↓vol.	~	~	~	↑T2	~	↑T2, ↓T1	~	~	no	~	~	~
	18.5	↑T2, ↓vol.	~	~	~	↑T2	~	↑T2, ↓T1	~	~	no	~	~	~
	23.5	↑T2, ↓vol.	~	~	~	↑T2	~	↑T2, ↓T1	~	~	no	~	~	~
9	29.0	↑T2, ↓vol.	~	~	↑T2	↑T2	~	~	~	~	yes	mild ↑T2: subcort., c.sem., cor.rad	~	~

### Genetic findings

**Fig. 1:**
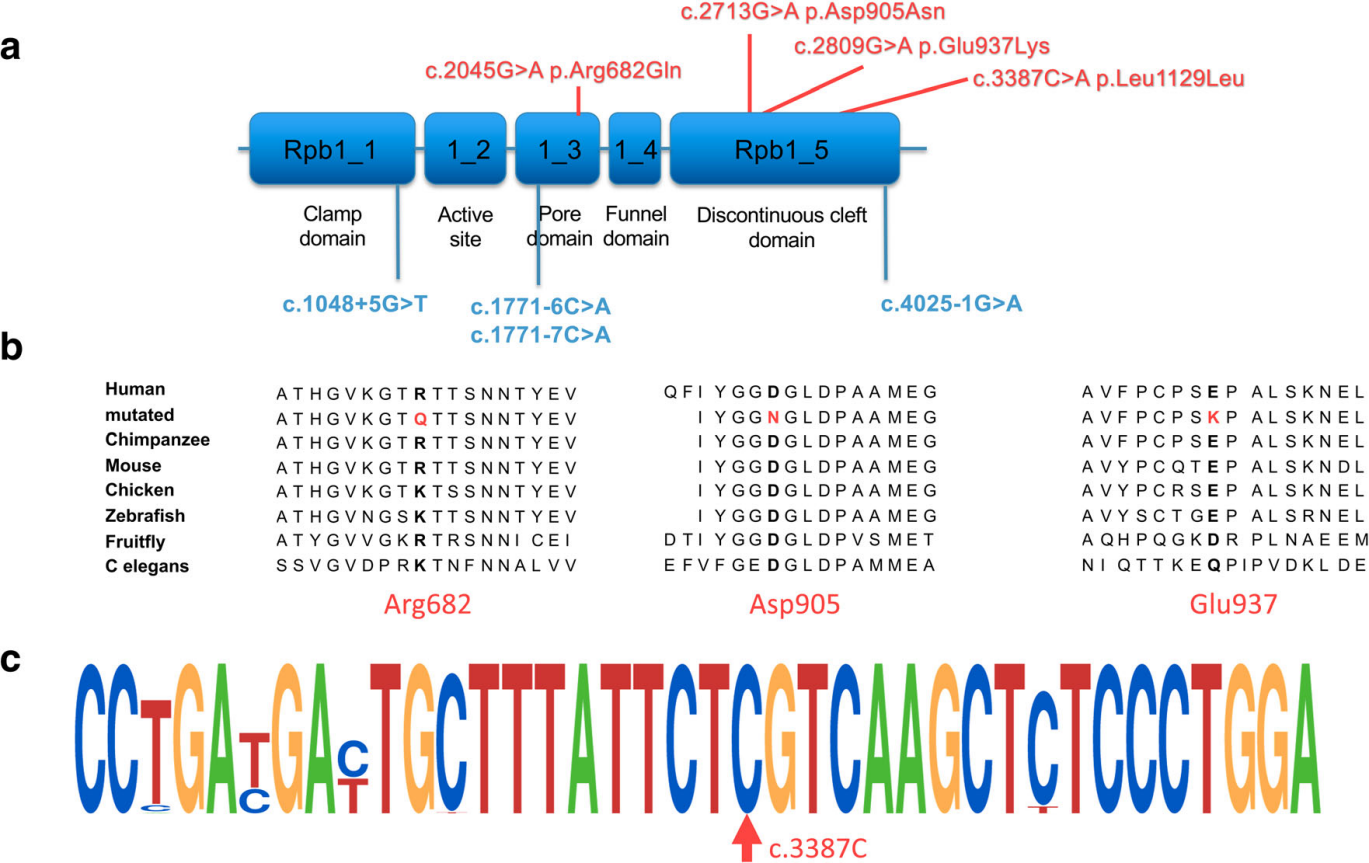
Localisation of variants and conservation in POLR3A. This figure shows the localization of intronic variants (in blue) and exonic variants (in red) in *POLR3A*. **a** Two missense variants are both located in the discontinuous cleft domain, one in the pore domain. **b** denotes conservation of mutated amino acids across different species. **c** Motifs of primary sequence conservation surrounding the c.3387C base pair based on alignment of 61 species using WebLogo, demonstrating the high conservation of the c.3387C base pair. The observed variant (c.3387C > A) does not lead to an amino acid change but is predicted to activate an exonic cryptic acceptor site in exon 26

All patients carried at least one of two intronic variants of *POLR3A*, c.1771-6C > G or c.1771-7C > G (Table 2; Fig. 1). While the two brothers were homozygous for c.1771-6C > G, all other patients were compound heterozygous: One patient carried the variant c.1771-6C > G, and six carried the variant c.1771-7C > G in combination with another variant. These were an intronic variant at/close to a splice site (*n* = 3; including one with c.1771-6C > G), a synonymous variant predicted to affect splicing (*n* = 1), and missense variants (n = 3). The two missense variants were both located in the discontinuous cleft domain and had not yet been described in patients. The c.2045G > A variant (heterozygous) has been described in a patient with classic 4H leukodystrophy [5]. The c.1048 + 5G > T variant in a homozygous state has been described in a patient with Wiedemann-Rautenstrauch syndrome [16] and compound heterozygous with c.1771-7C > G in a patient classified as spastic ataxia [12]. The c.1771-7C > G variant has been found in patients classified as spastic ataxia, in combination with a frameshift variant [11, 12]. The c.1771-6C > G variant has been described in patients in homozygous form with basal ganglia involvement [10]. The c.4025-1G > A, previously not reported in literature, affects a canonical acceptor site and can be considered as a loss-of-function variant.

### MRI findings

#### Basal ganglia

**Fig. 2:**
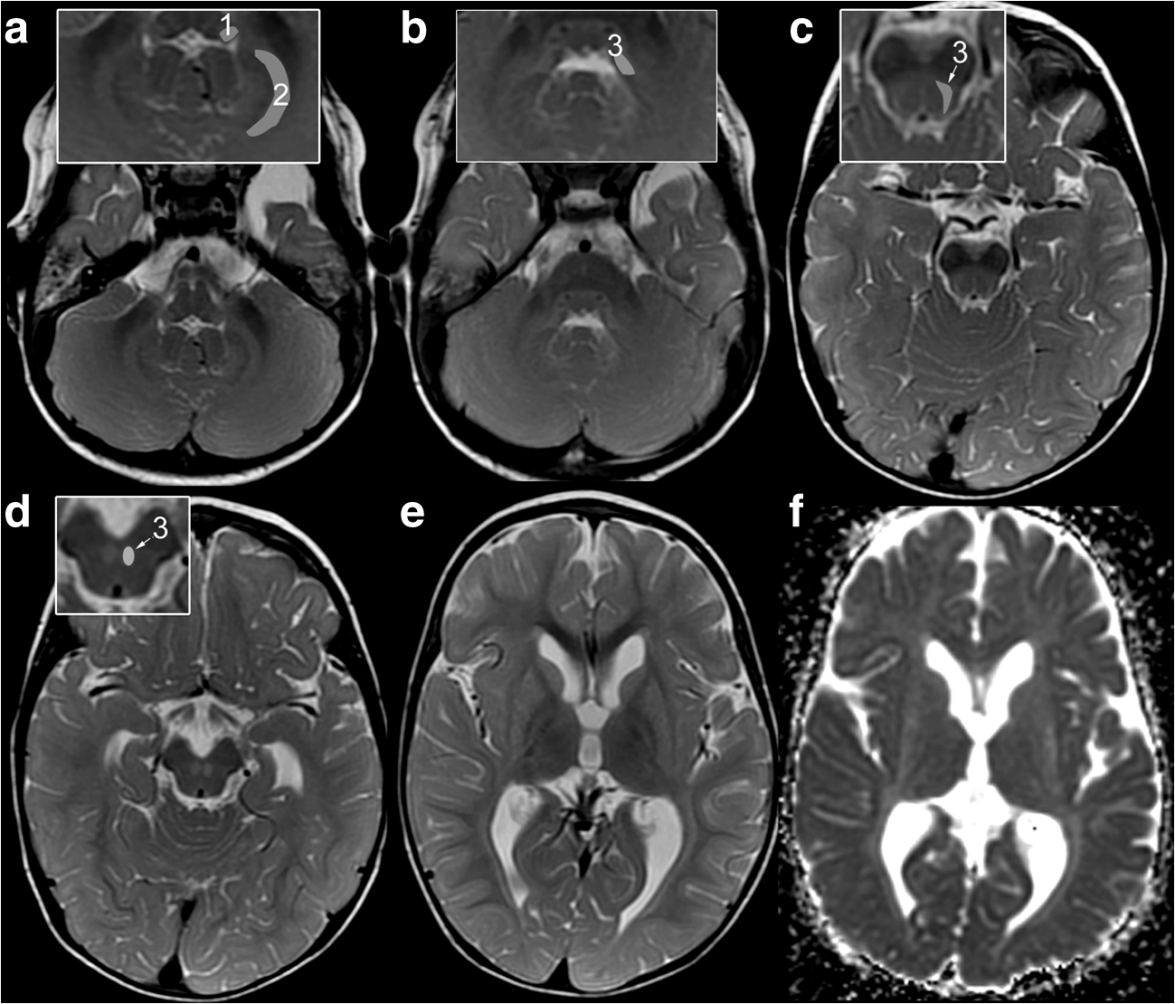
Characteristic MRI pattern of striatal variant of POLR3A-related disease. MRI in patient 1 at 1.5 years demonstrates the characteristic combination of atrophic, T2-hyperintense striatum ,and T2-hyperintense SCP (A-E: T2w; F: ADC-map; insets: 1 = ICP, 2 = peridentate white matter, 3 = SCP). **a** T2-hyperintensity of ICP (1) and peridentate white matter (2) are additional findings. **b** Further additional findings are T2-hyperintensity of tegmentum and intraparenchymal course of trigeminal nerve. T2-hyperintensity of SCP (3, insets in B-D) is seen along its course from the cerebellum (**b**)**,** dorsal mesencephalon (**c**) to the decussation in the anterior mesencephalon (**d**). **e**, **f**: Homogeneous, mild, and symmetric T2-hyperintensity of the striatum with volume loss and increased diffusion. NB the lateral medullary lamina between pallidum and putamen is commonly seen at this age due to its relative T2-hyperintensity compared with pallidum and putamen; increased conspicuity is due to T2-hyperintensity of putamen (**e**)

**Fig. 3:**
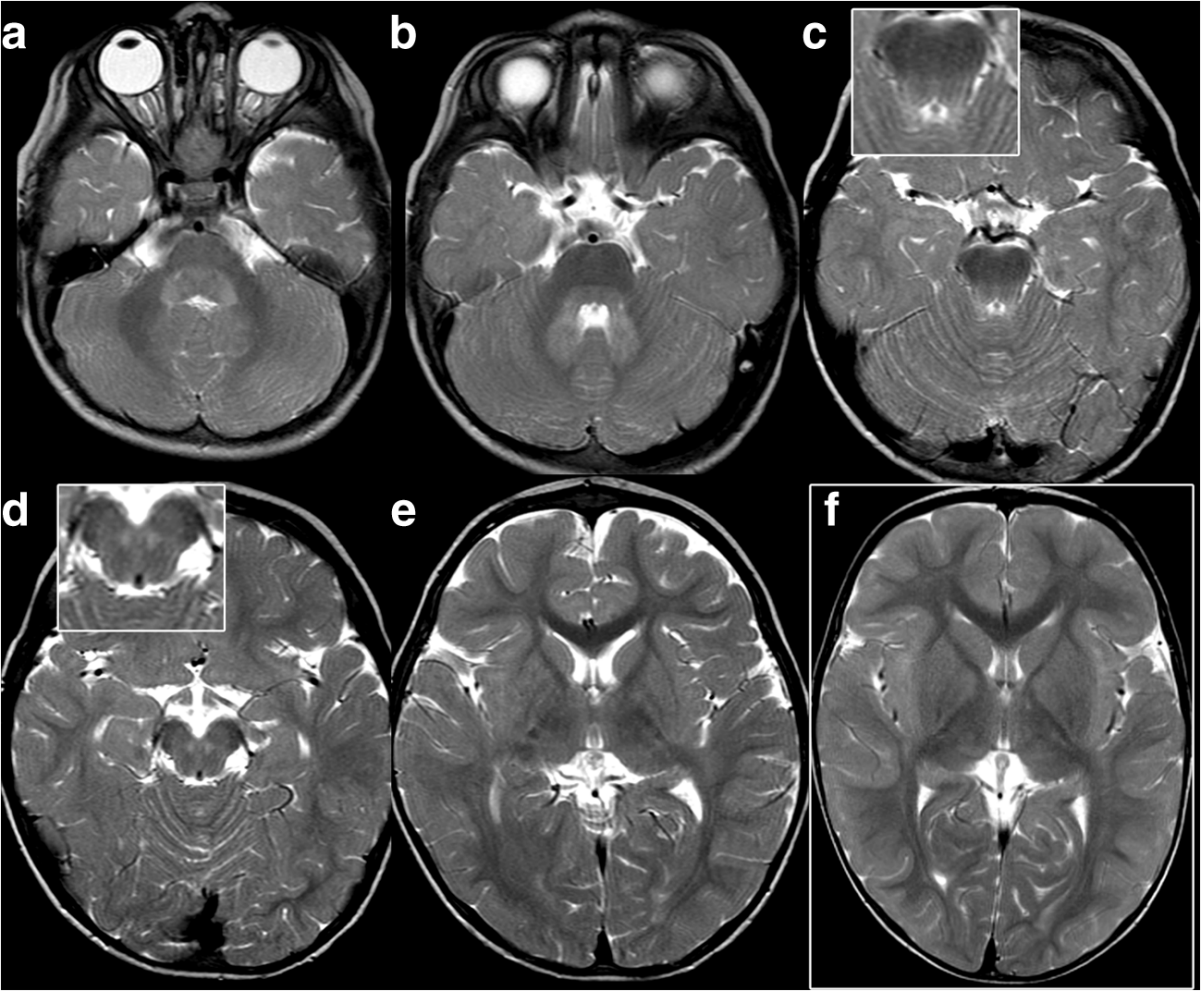
Small striatum and infratentorial changes at first MRI of patient 4. At 20 months, the striatum is small (**e**, age-matched control image in (**f**) for comparison), but its signal does not exceed that of the cortex and is normal. Note involvement of ICP (**a**), dentate nuclei, hila, and peridentate white matter (**b**), and of SCP including the decussation (**b**–**d**)

**Fig. 4:**
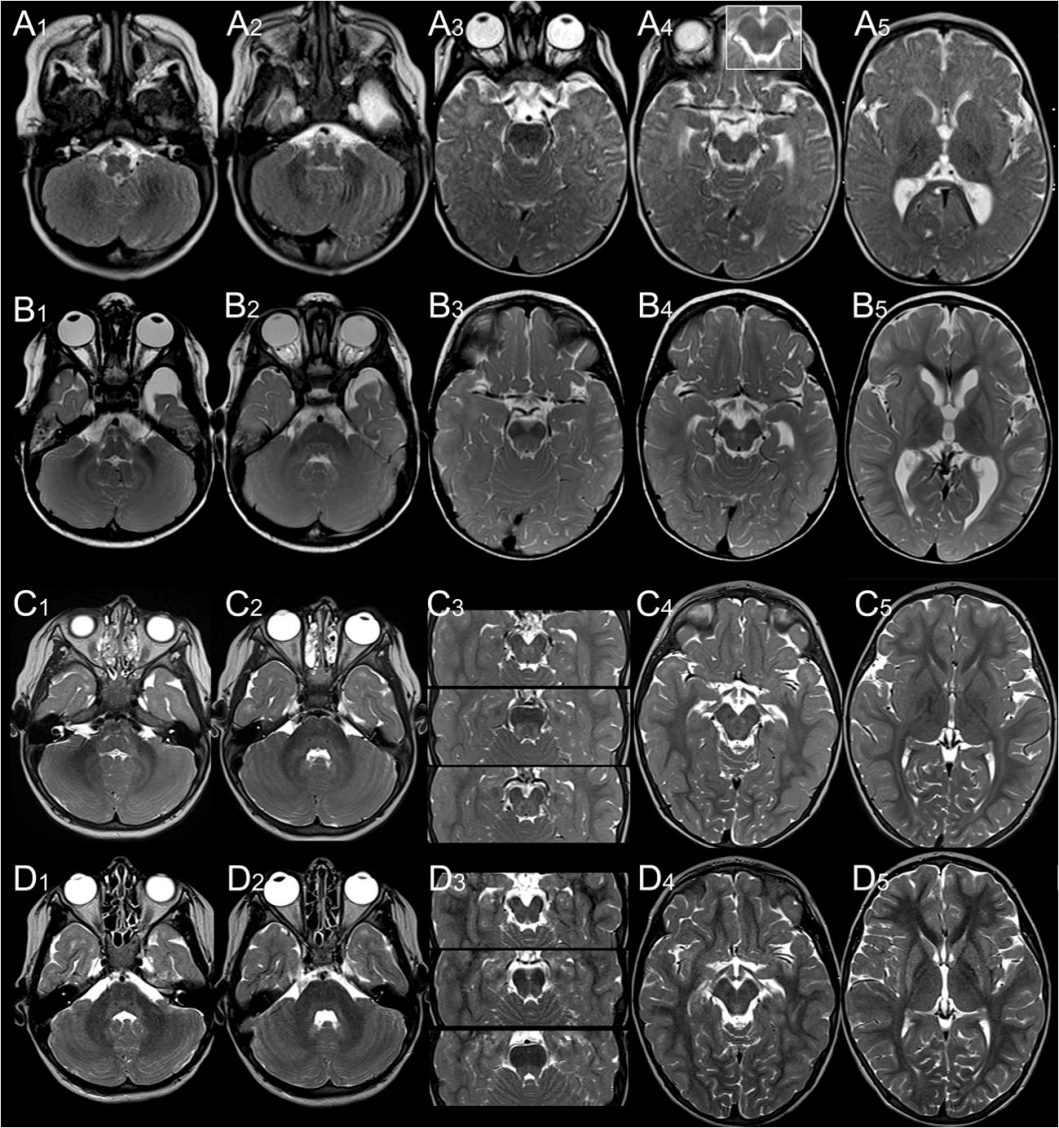
Evolution of brainstem and striatal changes in patients 1 (A, B) and 5 (C, D). **A**, In patient 1 at 0.5 years, the decussation of SCP mildly hyperintense (A_4_; inset: normal finding in age-matched control) and the striatum is normal (A_5_). **B** At 2 years ICP (B_1_), hila of dentate nuclei and peridentate white matter (B_1,2_) are newly hyperintense, and SCP is now clearly hyperintense along its mesencephalic course (B_3_) and in the decussation (B_4_). The striatum is homogeneously T2-hyperintense and atrophic (B_5_). **C** Patient 5 also has a normal striatum at 2 years (C_5_). ICP (C_1_), hila of dentate nuclei, peridentate white matter (C_2_) and SCP (C_3_), along to the red nucleus (C_4_) are T2-hyperintense. At 4.8 years (**D**), infratentorial changes (D_1–4_) are regressing whereas the striatum is newly T2-hyperintense and atrophic (D_5_)

Symmetric, homogeneous, mild T2-hyperintensity and atrophy of putamen and caudate nucleus (*striatum*) were present in all patients (Fig. 2), with corresponding hyperintensity on ADC maps and increased ADC (range 110–120 × 10–5 mm2/s) in those with diffusion-weighted imaging. Among the five younger patients with first imaging until 2 years, the striatum was already T2-hyperintense and atrophic in two patients at 0.9 and 1.0 year. In the other three, the striatum was initially small or normal and had become T2-hyperintense and atrophic only by follow-up at 1.5, 4.8, and 2.9 years, respectively (patients 4, 1, and 5; Figs. 3 and 4). The four older patients imaged between 4 and 29 years all had striatal T2-hyperintensity and atrophy (Table 3). In contrast, the striatum was normal in the 36 patients with classic 4H leukodystrophy re-reviewed for comparison.

The *pallidum* was not abnormally T2-hyperintense in our patients. In those five patients with gradient echo/susceptibility-weighted images and in the CT scan of patient 8, there was no indication of abnormal calcification, iron deposition, or blood degradation products.

#### Cerebellum and brainstem

All patients had signal alterations of the superior cerebellar peduncle (SCP) in at least one MRI, involving the decussation in seven patients and reaching the red nucleus in two of these. Additional findings were involvement of the hila of the dentate nuclei, the dentate nuclei, and/or the peridentate white matter in six, three, and four patients, respectively, and of the inferior cerebellar peduncle (ICP) in five. The middle cerebellar peduncles (MCP) were involved in none. In contrast, MCP were T2-hyperintense in 29, decussation of SCP in two, and ICP was not involved in any of the 36 patients with classic 4H leukodystrophy re-reviewed for comparison.

**Fig. 5:**
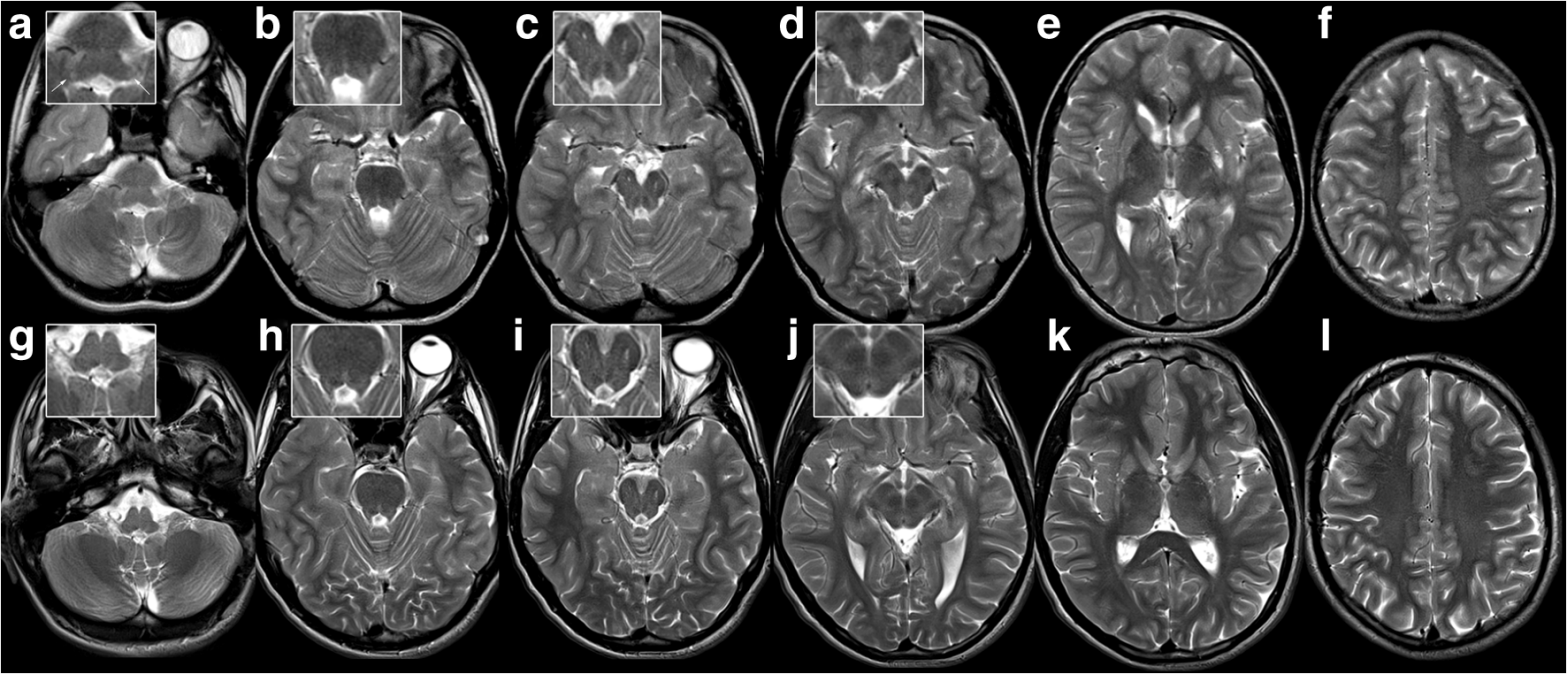
Striatal injury, regressing infratentorial changes, and normal supratentorial white matter in patient 7 at 13.6 and 18.5 years. T2-hyperintensity of inferior cerebellar peduncle (**a,** arrows in inset) and outlining the mesencephalic course of superior cerebellar peduncles (**b**, **c**; NB wide perivascular spaces in anterior mesencephalon) including their decussation (**d**) at 13.6 years (**a**–**f**). This has resolved by 18.5 years (**g**–**l**). The striatum is shrunken and T2-hyperintense (**k**), supratentorial white matter normal (including ADC, not depicted).

T2-hyperintensity of SCP did not significantly change in patient 8 between 12 and 23 years, whereas it resolved in his brother between 13 and 19 years (Fig. 5). Similarly, T2-hyperintensity of SCP, hila of dentate nuclei, peridentate white matter, and ICP clearly decreased in patient 5 between 2 and 4.8 years (Fig. 4). Conversely, infratentorial changes increased in the youngest patient between 0.5 and 1.5 years: Only the decussation of SCP was hyperintense at 0.5 years, while, at follow-up, T2-hyperintensity involved the entire course of SCP as well as the hila of the dentate nuclei, peridentate white matter, and ICP (Fig. 5). Taken together, these findings suggest that signal alteration of SCP, ICP, and dentate area may be a transient phenomenon.

#### Supratentorial white matter

Signal of supratentorial white matter on T2w and T1w images was normal in three of the four older patients examined between 4 and 29 years (patients 6–9), while mild T2-hyperintensity of pyramidal tract in the centrum semiovale and subcortical white matter was present in patient 9. The optic radiation was well myelinated, with T2w hyperintense signal in the surrounding white matter.

In the five younger patients with first imaging up to the age of 2 years, myelination was delayed and/or inhomogeneous with progressing, more advanced, or normal myelination for age outside of pyramidal and visual tracts: In the youngest patient (patient 1), myelination was delayed at 0.5 years. By follow-up at 1.5 years, myelination in the central region had not progressed, and there was new T2-hyperintensity in centrum semiovale, corona radiata, and visual tract, whereas myelination was normal outside of pyramidal and visual tracts (Supplemental Fig. 1). T2-hyperintensity of pyramidal tract and/or optic radiation was also observed in patients 2–5, in patient 5 with incomplete myelination of subcortical white matter. Signal of the posterior limb of internal capsule, including the pyramidal tract, was normal in all patients and no patient had frank hypomyelination.

#### Pituitary gland, bulbi, atrophy, and spinal cord

The pituitary gland including T1-hyperintense signal of neurohypophysis was normally visualized on sagittal T1w images in all patients. No patients had clearly elongated bulbi as an indicator of myopia, often seen in classic 4H leukodystrophy. With regard to atrophy, corpus callosum was normal in eight patients on visual inspection and thin in one patient (patient 3). Cerebellar atrophy was not observed. Spinal cord was normal in the three patients with spinal MRIs (patients 1, 5, and 6).

## Discussion

We present nine patients with biallelic variants in *POLR3A* carrying at least one of two intronic variants (c.1771-6C > G or c.1771-7C > G)*,* with predominantly extrapyramidal manifestations and characteristic MR changes of striatum, superior, and, often, inferior cerebellar peduncle. Their neurological presentation differs from classic 4H leukodystrophy, the initially described presentation of *POLR3A* variants. It also differs from a subform of spastic ataxia and the rare Wiedemann-Rautenstrauch syndrome (OMIM#264090), which have only recently been associated with *POLR3A*. Clinical presentation of these nine patients forms a continuum between a severe, extrapyramidal movement disorder with early onset at one end and juvenile parkinsonism with onset in childhood at the other end of the spectrum. Interestingly, six of the nine patients had abnormal dentition comparable with the abnormal dentition seen in 4H leukodystrophy. There was no evidence for endocrine involvement, although only three patients were old enough to exclude delayed puberty due to hypogonadotropic hypogonadism. Severe myopia, which occurs very frequently in children with 4H leukodystrophy and especially *POLR3B* variants, was not present.

Although our patients share variants with the spastic ataxia cohort (c.1771-7C > G; homozygous in several patients [12]), none was clinically classified as spastic ataxia. Interestingly, the original description of the patients homozygous for this variant also mentions extrapyramidal features and early onset of disease. And, while striatal changes are not mentioned, FLAIR-hyperintensity along the superior cerebellar peduncles was noted in almost all patients with the c.1909 + 22G > A variant, but, interestingly, not in the two patients homozygous for the c.1771-7C > G variant [12]. They might thus also be classified as the striatal variant of *POLR3A-*associated disease. One variant seen in our cohort, c.1048 + 5G > T, has also been found in spastic ataxia [12] and Wiedemann-Rautenstrauch syndrome [16]. However, our patient did not have the intrauterine and marked postnatal growth retardation, lipodystrophy, or distinctive facies characteristic of the progeroid syndrome of Wiedemann-Rautenstrauch [17].

The two brothers (patients 7 and 8) with the previously described, homozygous c.1771-6C > G variant [10], had a presentation similar to that of the three initially described patients [10] with onset in childhood and severe dysarthria, hypokinesia, and rigidity. A prominent, slow resting, and acting tremor, also sometimes called rubral tremor, in addition to severe dysarthria was seen in the older brother and in patient 9, who carried the c.1771-6C > G variant in combination with a missense variant.

The c.1771-6C > G variant was also described in one patient said to have spastic ataxia but also with dystonia [10, 11], without detailed MRI information. In a publication on atypical radiological findings in 4H leukodystrophy, one patient also carried this variant in heterozygous form, and, in retrospect, his last MRI showed small caudate and putamen with elevated T2 signal in addition to the signal abnormalities in the posterior limb of the internal capsule, fitting with his prominent extrapyramidal symptoms [9]. Recently, a young child with the c.1771-6C > G variant in trans with a frameshift variant has been published; the MRI shows the typical basal ganglia involvement described in this work, although this was not recognized as abnormal [18].

Among the other six patients, clinical manifestation varied despite sharing the c.1771-7C > G variant on one allele and the three youngest patients (patients 1–3) were much more severely affected than patients 4–6. The c.1048 + 5G > T variant on the second allele in one severely affected patient has been predicted to cause a frameshift with premature stop of translation [16]. It can be classified as a loss-of-function variant, similar to the variant found in patient 2 (c.4025-1G > A). The c.1771-7C > G variant itself has been shown to lead to two aberrant transcripts in addition to the normal cDNA, interpreted as activating a leaky splice site with both wild-type and aberrant transcripts [12]. Similar results were obtained for the c.1771-6C > G variant, with skipping of exon 14 and a premature termination of a part of the transcripts, with the shorter transcript being subject to nonsense-mediated decay [10].

Findings at brain imaging reflect the prominent extrapyramidal movement disorder: T2-hyperintensity and atrophy of the striatum were present in all patients, either at first imaging or on follow-up. In one case, a small striatum preceded T2-hyperintensity. A normal striatum was not seen after onset of extrapyramidal movement disorder. T2-hyperintensity was discrete and relatively inconspicuous compared with striatal injury, e.g. in glutaric aciduria type 1 or ischemia. Atrophy varied between mild and severe, e.g. in patients 1 and 7 (Figs. 2 and 5), similar to the initially described patients with striatal injury and homozygous c.1771-6C > G variant [10]. Extrapyramidal signs can also develop in classic 4H leukodystrophy [6], with visually normal basal ganglia on brain MRI.

The second characteristic MRI feature was involvement of SCP, which was present in all our patients. This included the dentate nucleus and/or its hilus as the starting point of the efferent neurons of SCP in six patients and the red nucleus as a relay station in two of these. ICP was additionally involved in six patients. Involvement of SCP in patients with *POLR3A* variants has previously been reported for the spastic-ataxia cohort [12] and in four of eight atypical patients [19]. It is also depicted in a report of a patient with hypomyelination and a previously unreported homozygous variant of c.2423G > A in exon 18 (Fig. 2a in [20]). Involvement of the red nucleus as a relay station of SCP was reported for the three patients homozygous for the c.1771-6C > G variant [10]. The symmetric, anterior mesencephalic T2-hyperintensity also reported might rather correspond to the superior mesencephalic course of SCP than the proposed intraparenchymal course of the oculomotor nerve [10].

Changes in SCP, dentate and red nuclei, and ICP were not clearly associated with striatal injury since they preceded striatal injury in two patients and were subsequent in one. Moreover, their decrease and disappearance in two patients suggest that they are a potentially transient phenomenon. While SCP involvement in the spastic-ataxia cohort was thought to represent the structural correlate of the cerebellar manifestation [12], contribution of SCP and ICP to the clinical picture in our patients is difficult to pinpoint. This is due to the predominantly extrapyramidal movement disorder, the infrequent ataxia, and the unchanged presentation in those patients with decreasing or resolving changes, although the prominent tremor in patients 8 and 9 is certainly compatible with the involvement of the striatum and the dentate outflow tract [21].

Compared with classic 4H leukodystrophy, infratentorial involvement was practically inverted in patients with the striatal variant of *POLR3A*-related disease: While MCP was normal in the striatal variant, T2-hyperintensity of MCP has been noted in reported cases of 4H leukodystrophy [22–24] and was present in 29 of the 36 the previously reported patients with classic 4H leukodystrophy [7] re-reviewed for comparison. SCP was involved in only two patients with classic 4H leukodystrophy, but in all patients with the striatal variant, ICP in none of the patients with classic 4H leukodystrophy and in six of nine patients with the striatal variant *POLR3A*-related disease. Moreover, T2-hyperintensity of cerebellar white matter with relatively T2-hypointense dentate nucleus and early cerebellar atrophy are common in 4H leukodystrophy [25], while cerebellar signal changes in patients with the striatal variant were restricted to dentate area, and cerebellar atrophy was absent. In addition, none of the 36 patients with classical 4H leukodystrophy re-reviewed for comparison had striatal T2-hyperintensity.

In 4H leukodystrophy, diffuse hypomyelination is a core finding, commonly with some myelination of the visual tract, the pyramidal tract in the posterior limb of the internal capsule, and the anterolateral thalamus [5, 25]. In contrast, myelination delay and white matter changes in our patients preferentially involved the optic radiation and pyramidal tracts, and none had frank hypomyelination. Thinning of corpus callosum, another common feature of 4H leukodystrophy, though somewhat less common in patients with carrying *POLR3A* variants [5], was only present in one of the nine patients with the striatal variant.

In conclusion, we present nine patients with biallelic variants in *POLR3A* carrying at least one of two intronic variants and prominent extrapyramidal involvement. MRI is characterized by striatal injury, involvement of SCP and commonly of ICP, and, variably, irregular myelination of pyramidal and visual tracts. Although our study is limited by the relatively small number of patients, clinical manifestation and MRI differ from 4H leukodystrophy and are consistent with a distinct, striatal variant of *POLR3A*-related disease.

Recognition of the characteristic MRI pattern, including awareness of the potentially relatively mild T2-hyperintensity and atrophy of the striatum, should trigger genetic testing for *POLR3A* in patients with unexplained extrapyramidal movement disorders.

## Electronic supplementary material

ESM 1 (PDF 2589 kb)
