# Supplementary material for: POLR3A variants with striatal involvement and extrapyramidal movement disorder
Source: Neurogenetics. 2020 Jan 15;21(2):121–33. doi: 10.1007/s10048-019-00602-4 (PMC7064625; doi:10.1007/s10048-019-00602-4)
Supplement: Supplementary file 1 — (PDF 2589 kb) [file 10048_2019_602_MOESM1_ESM.pdf]

Supplemental material

Case descriptions

Patient 1:

This patient is currently 2 years old. Pregnancy was normal, delivery and neonatal period were uneventful. She did not start smiling, and from the age of 2 months on she failed to thrive. She was irritable and vomited frequently; these symptoms were partly alleviated by the use of a proton pump inhibitor. She learnt to grasp and to transfer from one hand to another at 5 months, but lost this at age 7 months. From age 12 months on she had problems with clearing mucus. She was uncomfortable but slept well. Her contact improved around the age of 12 months. From the age of 15 months on, parents noted muscle jerks. Her dentition was abnormal; the first teeth to erupt were her lowed median incisors, then her upper canines. At the age of 17 months, she did not yet have upper incisors. Neurological examination at 17 months showed a restless child who made eye contact and was able to follow objects with saccadic pursuit. There was a fine rotatory nystagmus. She had reasonable head balance, no voluntary movements, intermittent opisthotonic posturing with extended and endorotated arms and fisting and scissoring of her legs. In the short quiet periods, there was no catch, muscle tendon reflexes were present, but not exaggerated and plantar responses were flexor. EEG showed frequent polyspike and polyspike-slow wave complexes, once with a myoclonic jerk. Treatment with levetiracetam had good effect on alertness and frequency of myoclonic jerks. She still had failure to thrive.

Patient 2:

This currently 2-year-old girl is the first child of healthy, unrelated parents. Family history was unremarkable, pregnancy, delivery, and perinatal period were normal, but her mother did note that her daughter did not cry or scream. Some head control was achieved at age 8 weeks, and she was able to grasp her own hands and to put them into her mouth. At this time general muscular hypotonia became obvious. At 3 months of age she did not show social interaction, neither smiling nor following with her eyes. She had a uniform and noisy breathing pattern. By the age of 4 months she had lost head control, her spontaneous movements decreased and had become stereotypic, undirected and dystonic. This movement disorder worsened over time. She developed a pes equinus on both sides. At 5 months of age she showed a preferentially right-sided head position, axial hypotonia, with intermitted opisthotonus, and brisk muscle tendon reflexes. At that time she had lost the ability to put her hands in her

mouth. Swallowing and feeding difficulties from age 6 months on led to failure to thrive and required placement of a nasogastric tube at 11 months of age. Problems of swallowing her saliva required continuous evacuation with a suction device and oxygen supply during the night. Dentition was severely delayed and started only after the age of 14 month with molar teeth as the first teeth to erupt.

Patient 3:

This patient, 21 months old, presented in the neonatal period. Pregnancy and delivery were uneventful, but parents noted abnormal movements right from the beginning. Visual contact was difficult, and she smiled only rarely. Her motor development was delayed; she started to roll over around the age of 12 months. She failed to thrive and was repeatedly admitted with respiratory tract infections. At the age of 13 months, she was diagnosed with pneumococcal meningitis and developed bilateral subdural effusions, necessitating evacuation. Neurological examination showed a child who made very short visual contact, with axial hypotonia and continuous choreic movements of her whole body. She tried to grasp objects, again with choreic movements. She had some head balance. Muscle tendon reflexes were normal, plantar response was indifferent. She never had seizures; her EEG showed a reasonable background activity. Brainstem evoked auditory potentials (BEAR) were abnormal, with reproducible waves I and II and absent wave V. Due to feeding problems, a nasogastric tube had to be placed at age 18 months. She had abnormal dentition with lack of maxillary incisors at age 16 months, while the upper canines and mandibular incisors were present.

Patient 4:

This patient, currently 7 years old, was born at term after a pregnancy notable for oligohydramnion but otherwise uneventful. Sucking difficulties were present since the neonatal period with failure to thrive from age 6 months on. His development was delayed since the first months of life: Head control was achieved at 4 months, social smiling began at 4-5 months, crawling at 13 months and at 22 months he had an unsteady gait. There was absence of expressive language before 3 years of age despite having a communicative interest. Neurological examination at the age of 3 years revealed drooling, slow movements, brisk deep tendon reflexes and unsteady wide-based gait. At the age of 5 years, he had excessive blinking, suggestive of blepharospasm and a spastic-dystonic tetraparesis with brisk deep tendon reflexes, ankle clonus and indifferent plantar reflex. He continued having trunk instability and global bradykinesia. He was able to speak few words but was difficult to

understand. Cognition was never formally tested but felt to be subnormal. His dentition was abnormal, with delayed eruption of maxillary incisors.

Patient 5:

This patient, currently 7 years old and born at term after an uneventful pregnancy, had a mildly delayed development. She was able to sit at the age of 10 months and to walk at the age of 22 months. Language development was also delayed (first words at the age of 18 months, 2-word-sentences at the age of 3 years). From the age of 4.5 years on, parents noted that she fell more frequently than usual and that her speech became more difficult to understand with a stutter. At the age of 5.5 years, ataxia was apparent with relatively preserved fine motor skills. She could walk without support, albeit with a broad base. Her eye movements were normal, there were no pyramidal signs. At the age of 6 years, these findings were unchanged barring a spontaneous extension of her left big toe. Dentition was normal. She had mild myopia. Growth was normal.

Patient 6:

This patient is currently 6 years old. He was born after a normal pregnancy via induced vaginal delivery at 41 weeks and vacuum extraction due to a non-reassuring fetal heart rate during labor Apgar scores were 2-5-8 at minute 1, 5 and 10, respectively. He needed oxygen and positive pressure ventilation and was admitted to the neonatal intensive care unit for one day for a self-limiting pneumothorax. The patient was discharged from the hospital at 4 days of life. He had mild jaundice which did not require treatment. There were no other perinatal complications. The parents first became concerned with the patient's development around age 13 months because he tended to walk on the inside of his feet. He never had any frank episode of regression. The neurological examination at the age of almost 5 years revealed mild to moderate sialorrhea as well as moderate dysarthria. Cranial nerve examination demonstrated saccadic pursuit, hypometric saccades and uncoordinated tongue movements. Motor examination revealed mild axial hypotonia, moderately increased tone in the upper extremities and mildly increased tone in the lower extremities, grossly normal strength and normal muscle bulk. Rapid alternating movements of the upper and lower extremities were slower than expected (attributed mainly to dystonia) and variable in amplitude and rhythm (cerebellar) with overflow of the contralateral hand and the feet. Finger-to-nose showed intention tremor. Finger chase was dysmetric. His gait was broad based with a combination of dystonia and to a lesser extent spasticity. Posturing of the hands was present when

walking. Tandem gait was unstable and could only be performed with support. He was unable to jump. He could stand on one foot only with support. His tendon reflexes were all brisk and his toes were upgoing bilaterally. Growth was normal. He had mild speech delay, associated with dysarthria. At the age of 6 years, his gait is still unstable. The patient has no history of dysphagia. His dentition is normal and he is not myopic.

Patient 7:

This patient, aged 22 years, is the second child of consanguineous Turkish parents. Pregnancy and delivery were normal. His early development was unremarkable; he could walk without support at age 12-13 months. At the age of 4 years, psychomotor retardation was first noticed. From the age of 6 years, his motor function slowly deteriorated. This was somewhat accelerated during mild infections and puberty. At the age of 9 years he changed from a mainstream school to a school for children with special needs due to learning disability. Formal cognitive testing was not performed. Neurological examination at age 21 years showed normal consciousness. He had poor facial expression and dysarthria. Eye movements showed saccadic pursuits. Muscle bulk was decreased. There was no tremor, but dysdiadochokinesis. He had a shuffling gait, with festination and postural instability. Muscle tendon reflexes were normal. He had spontaneous extension of the big toe, interpreted as striatal toe. He showed dysarthria. Dentition and puberty development were normal. He had mild hyperopia.

Patient 8:

This patient, the older brother of patient 7, is currently 26 years old. As in his brother pregnancy, delivery, and early development were unremarkable. He could sit without support at age 9 months and walk without support at age 15 months, spoke his first words at age 18 months and 3-word sentences around the age of 25 months. He was always difficult to understand. At age 3 years, developmental delay was noticed, in particular of speech. From age 5 years onwards, the patient slowly deteriorated. He developed anarthria and severe dysphagia, he mainly communicated via written messages on his mobile phone. He had severe hand tremor. He was still able to walk, but used a wheelchair and, for longer distances, an electric wheelchair, and suffered from frequent falls. His IQ in primary school was between 70 and 80; he visited a school for children with special needs from the 1st class. The restricted possibilities of communication made assessment of his cognitive abilities difficult and while there seemed to be some cognitive decline, there were also concerns that he was

not sufficiently challenged at his school. His dentition was abnormal with the upper incisors appearing first; he still had 2 persisting decidual teeth, 31 and 41. Puberty development was normal. Neurological examination at age 26 years showed normal consciousness. He communicated effectively with gestures. His eye movements were remarkable for saccadic pursuit and hypometric saccades. Muscle bulk was decreased. He had poor facial expression, his mouth was mostly open. Tongue movements were severely impaired. He had a resting tremor of 3/sec, and also tremor with movements. He needed several attempts to get up from a chair. Postural instability, festination and freezing were all present. Muscle tone was severely elevated in arms and legs without catch, tendon reflexes could be elicited. There was a striatal toe.

Patient 9

This patient, currently 29 years old, was born after a normal pregnancy and an uneventful delivery. Her family history shows hypertension on the father's side and myopia on the mother's side. The parents first noted abnormalities when the patient began walking at 14 months of age: She was abnormally prone to falling and walked with an unusual gait where her torso increasingly leaned forward with every stride until she lost her balance. Her mobility and balance problems gradually worsened. At 8 years of age, her speech started to deteriorate and at age 12 years she became wheelchair dependant and needed assisted communication. She also developed jerky movements of her arms. The patient suffers from progressive dysphagia. She has no history of respiratory infections. Dentition was abnormal in both timing and order with the molars the first teeth to erupt and the upper and lower incisors only erupting at 4 years of age. Menarche was normal at age 11 years. The patient has mild myopia. At the most recent neurological examination, understanding of language was entirely normal. The patient was able to speak several words, with severe dysarthria, and communicated mostly with a computer. Eye movements were notable for saccadic pursuit and gaze-evoked nystagmus. She kept her arms close to her abdomen and at times behind her back, in order to minimize tremor and involuntary movements. The arms showed a resting tremor (frequency 3/s) which worsened into a coarse tremor with mild posturing of the fingers with action, compatible with so-called rubral tremor. There was no increased muscle tone of the arms or legs barring the ankles which also showed Achilles tendon contractures. Tendon reflexes of the arms were easily elicited and the Achilles tendon reflex showed bilateral sustained cloni. Both plantar reflexes were extensor. She had mild myopia, recently diagnosed.

Supplemental Figure

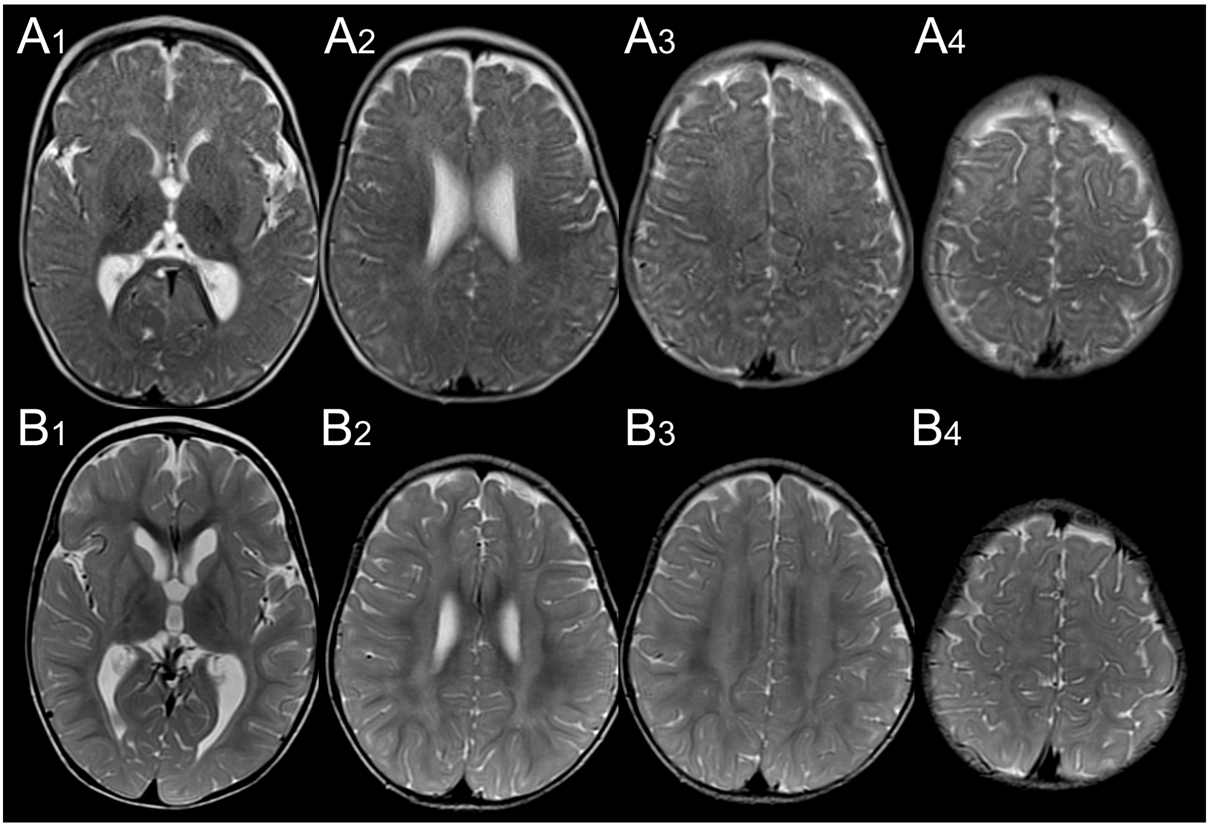

White matter changes in patient 1:

A: Myelination in patient 1 is mildly delayed at 5 months with iso- instead of hypointensity of pyramidal tract in corona radiata to cortex on T2w images (A_2) and very little T2-isointensity in the central region (A_4); NB the striatum is normal (A_1). B: At 2 years myelination outside pyramidal and optic tract has progressed and is adequate for age, while there is new T2-hyperintensity of the optic radiation (B_1) and of the pyramidal tract (B_{2-4}) and beyond the pyramidal tract in corona radiata and centrum semiovale. NB the striatum has become homogeneously T2-hyperintense and atrophic (B_1).
